# Towards symbiotic approaches between universities, sustainable development, and cities

**DOI:** 10.1038/s41598-022-15717-2

**Published:** 2022-07-06

**Authors:** Walter Leal Filho, Liliana Caughman, Maria Alzira Pimenta Dinis, Fernanda Frankenberger, Anabela Marisa Azul, Amanda Lange Salvia

**Affiliations:** 1 Department of Natural Sciences, Manchester Metropolitan University, Chester Street, Manchester, M11 5GD UK; 2 Research and Transfer Centre “Sustainable Development and Climate Change Management”, Hamburg University of Applied Sciences, Ulmenliet 20, 21033 Hamburg, Germany; 3 Postdoctoral Research Scholar, Earth Systems Science for the Anthropocene, Arizona State University, Tempe, USA; 4 UFP Energy, Environment and Health Research Unit (FP-ENAS), University Fernando Pessoa (UFP), Praça 9 de Abril 349, 4249-004 Porto, Portugal; 5 Business School PUCPR, Pontifical Catholic University of Paraná, Rua Imaculada Conceição, Curitiba, 1155 Brazil; 6 Brazil Business School, Universidade Positivo, Rua Pedro Viriato Parigot de Souza, Curitiba, 5300 Brazil; 7 CNC-Center for Neuroscience and Cell Biology, University of Coimbra, 3004-504 Coimbra, Portugal; 8 CIBB-Center for Innovative Biomedicine and Biotechnology, University of Coimbra, Coimbra, Portugal; 9 IIIUC-Institute for Interdisciplinary Research, University of Coimbra, 3030-789 Coimbra, Portugal; 10 Graduate Program in Civil and Environmental Engineering, University of Passo Fundo, Campus I - BR 285, São José, Passo Fundo, RS 99052-900 Brazil; 11 European School of Sustainability Science and Research, Hamburg University of Applied Sciences, Ulmenliet 20, 21033 Hamburg, Germany

**Keywords:** Environmental sciences, Environmental social sciences

## Abstract

Universities are key actors and play a central role in the cities which host them, either as employers, consumers or simply as a magnet to young people and cultural activities. They can be also influencers and supporters of cities in the field of sustainable development. Through an exploratory method and aiming to explore the efforts deployed to foster closer links between universities, sustainable development, and cities and address a literature gap in this regard, this study considers the contribution of universities to sustainable development at the city level. Based on some of the ongoing initiatives worldwide and an international online survey documenting measures undertaken, this study translates the commitment to pursuing sustainable development within cities, with responses from a sample of 45 countries. This study lists some items which may help foster more symbiotic relations between cities and universities. The findings of this study may be used as baselines for strengthening the connections between universities and cities in addressing the sustainable development challenges, as demonstrated through the responses obtained. Accordingly, some suggested actions involving cooperation may include increased communication with city stakeholders and the undertaking of joint initiatives and projects, taking advantage of the ongoing sustainable development challenges worldwide.

## Introduction

It is widely acknowledged that universities are key actors in the development of the regions in which they are located ^1^. They have a symbiotic connection to residential, commercial, and industrial areas. Information and technology developed in a university context, and extended to society, have the potential to influence the decision-making process of individuals towards sustainability ^2^. Through scientific research cooperation, countries like China are distributing knowledge through higher education resources sharing, aiming to raise talent and establish high-tech research and development ^3,4^, thus promoting regional sustainable development, and contributing to global economic development and sustainability through universities. Other countries such as the case of Vietnam, where universities are not used to full engagement in the local community ^5^, have started to invest in knowledge as potential drivers to advance sustainable development, aiming to spread knowledge and improve the human condition. Malaysia is another example engaging in sustainable development towards society change ^6^, with sustainability being increasingly perceived as important in the context of the university-society connection, contributing to intensifying environmental awareness, with major positive impacts on society and economic development. Despite previous evidence in a changing world addressing sustainable development change through cooperative means at the local level, the mutual influence on the interconnection between universities and cities is not always considered ^7^. The collaborative problem-solving competency of students, highlighted by the Organization for Economic Cooperation (OECD) as a key competence in the twenty-first century plays an important role in the context of sustainable development, through preparing students for a job as well as learning for life, in the context of Industrial Revolution 4.0 ^3,8^, an example of how universities may strongly influence society towards sustainable development achievement. This mutual cooperation is seen as evidence of civilization and playing a major role in the urban economy, contributing to a better society through teaching, learning, and civic engagement. Thus, universities have the ability to engage with multiple players in communities, in order to co-create and transfer knowledge and innovation, foster local economies and promote cultural diversity ^9^. There are many advantages of having a university in a city, but the major plus point is that they promote economic prosperity, alongside social inclusion and development. Universities are large employers, and therefore provide jobs to hundreds of people in their cities. They also support many small companies, where students have part-time jobs. The latter also bring money to the cities since they need to buy products and services. The contribution of universities are wide-ranging, encompassing scientific and technological advances and innovation in a wide range of fields, from digitalisation to energy provision, mobility and infrastructure, food security and landscaping, active lifestyle, and human well-being, with all these aspects encompassing sustainable development.

The nature and extent of universities’ local impacts are related to the type of collaboration that they are involved in Ref.^9^, especially in respect of economic and cultural development ^10^. Universities are engaged in income-generating activities and strategies and technology transfer, which include commercialising ideas and inventions that are developed through research. Revenues are mainly generated through licensing agreements and sponsored contracts, which often generate revenue that is a welcome support for local development ^11^. Therefore, both national and international students not only serve the purpose of strengthening universities in respect of learning and teaching but also help to support the local economy and culture. This, in turn, helps to increase their contribution to social sustainability ^12^.

Furthermore, universities play a key role in ensuring a transformative innovation in terms of local sustainable development ^13^. This is either carried out through the incorporation of sustainable development in the curricula or via community-based initiatives ^14^. Universities can also promote lifelong learning and teaching, which may allow the pursuit of local development on sustainable development ^15^.

Departing from some scarcity of literature on the topic being addressed, this paper attempts to contribute to the knowledge on universities’ connections with cities pursuing sustainable development through an exploratory study involving literature information collection and an online survey assessing the effort undertaken to collaborative joint work among universities and cities worldwide under the scope of sustainable development. The research question is: how can more symbiotic relations between cities and universities in a sustainable development context be pursued?

Based on a comprehensive instrument made available online, assessing the connections between universities, sustainable development, and cities, the originality of this study is that it focuses on potential links between cities and universities in a sustainable development context, filing a gap in the literature at this respect. The subsequent parts of this paper explore these connections, allowing us to point out the specific measures being undertaken in this context, taking full advantage of symbiotic collaboration opportunities between all stakeholders to advance sustainable development.

## The close relationships between universities and cities in a sustainability context

Universities, frequently regarded as one of the nuclei of surrounding communities ^5^, have a major role to plan in the field of sustainable development, especially due to their many connections and local involvement. This connection results in robust positive evidence for local economic performance, as a consequence of innovation, development of human capital, and support of democratic values ^16^. At the present, the relationship between universities and cities is beyond a knowledge-based economy to face the interdependence with the dimensions of sustainable development, namely social, economic and environmental. Because a distinctive feature of universities is that they foster creativity, innovation, social and intellectual development, and collaboration ^17,18^, they are able to transfer this more intensively to the environment than to the universities themselves. Thus, the opportunity to develop large-scale solutions transferable to society will result in the dissemination of innovation at a global level, with huge gains to the surrounding communities. An example of a large-scale solution is provided by the Massachusetts Institute of Technology Media Lab ^19^, whose innovation is used round the world.

There are some examples of initiatives where universities and cities have successfully cooperated on matters related to sustainable development. One of them can be seen in Portland, Oregon, USA, where the city government has been working with the Institute for Sustainable Solutions at Portland State University, to co-create actor-centric transformative capacity ^20^. The city and university work together to develop and implement two cross-disciplinary workshops that brought together city experts and academic researchers to explore climate impact scenarios. This collaboration led to the formation of a new city working group that continuously integrates academic knowledge and practices into urban planning focused on sustainable development and is accelerating progress towards goals ^20^. Other examples of fruitful cooperation showing some of the initiatives being carried out are summarized in Table 1 and illustrate the ongoing effort in successfully implementing symbiotic initiatives between universities and cities that contribute to achieving sustainability transformations, and positively impacting the society.

**Table 1:** Some examples of synergies between universities and cities in the context of sustainable development.

Case	Location	Synergies	Source
European School of Sustainability Science and Research (ESSSR) “Sustainability Sessions”	Hamburg, Germany	ESSSR works with the city of Hamburg, organising experts events attended by city staff	Leal Filho ^21^
University of Southern Santa Catarina “Driver of Sustainable Communities”	Santa Catarina, Brazil	Participative management between universities and municipalities empowering and educating the local community	Amorim et al. ^22^
City–University Partnerships (CUPs)	Lüneburg and Karlsruhe (Germany), Mexico City (Mexico), Portland, and Tempe (USA)	Mutual understanding of individual and collective competences at both the university and the city to transfer sustainability solutions	Withycombe Keeler et al. ^23^
The Co-production of Sustainable Future Scenarios	Phoenix, Arizona, USA	Co-produce visions and transition pathways of positive futures that develop and integrate interventions for sustainability transformations	Iwaniec et al. ^24^
Xiasha University Town and the contribution of Higher Education Institutions (HEIs) to regional development	Hangzhou, China	By developing university towns, HEIs contribute by forming a learning region and focusing on socio-economic development through skills, innovations, culture, and community	Mei and Symaco ^25^

**Figure 1:**
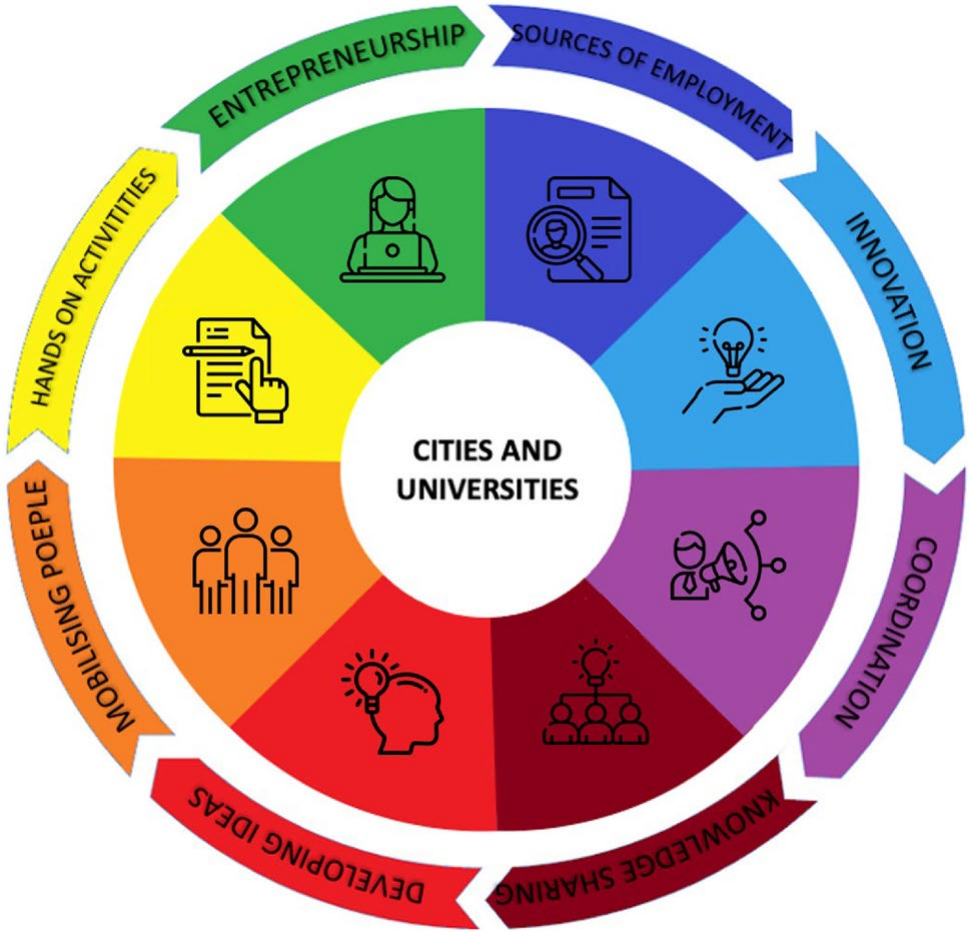
Synergies between universities and cities to achieve sustainable development (*Source* prepared by the authors).

The key synergies identified can be put into a framework that can support cities and universities in developing and implementing actions toward sustainable development, as presented in Fig. 1. The advantage of these synergies is that they may be accomplished at multiple levels, being able to suit the specific conditions of an organization and its settings.

## Methodology

This study pursued an exploratory methodological approach involving a convenience sampling data collection through an international online survey being shared among common networks and contacts, to investigate the contribution of universities to sustainable development at the city level. Based on previous extensive literature review, the comprehensive questionnaire developed focused on three main aspects of the cooperation between universities and local actors at the city level: (a) cooperation partners, (b) type of cooperation, and (c) challenges that hinder cooperation, following Leal Filho et al. ^26^ and other current examples of cooperation between universities and cities, reported previously. With one close-ended question per aspect, the lists of options were based on the most common agents: (i) city government, local companies, local non-governmental organizations (NGOs), and teaching/research institutions; (ii) interactions: joint events, joint projects, and internships from students in the organisations; and (iii) challenges: Lack of time to invest on cooperation, lack of interest from local counterparts, lack of interest from the university, lack of local contacts to cooperate, and lack of interest to cooperation on sustainable development as observed in the literature. An open space was also provided in each question for respondents interested in sharing additional responses. Based on the perception of the respondents, this exploratory study also assessed the current level of emphasis given to the *cooperation* of each university with city organisations on matters related to sustainable development.

**Figure 2:**
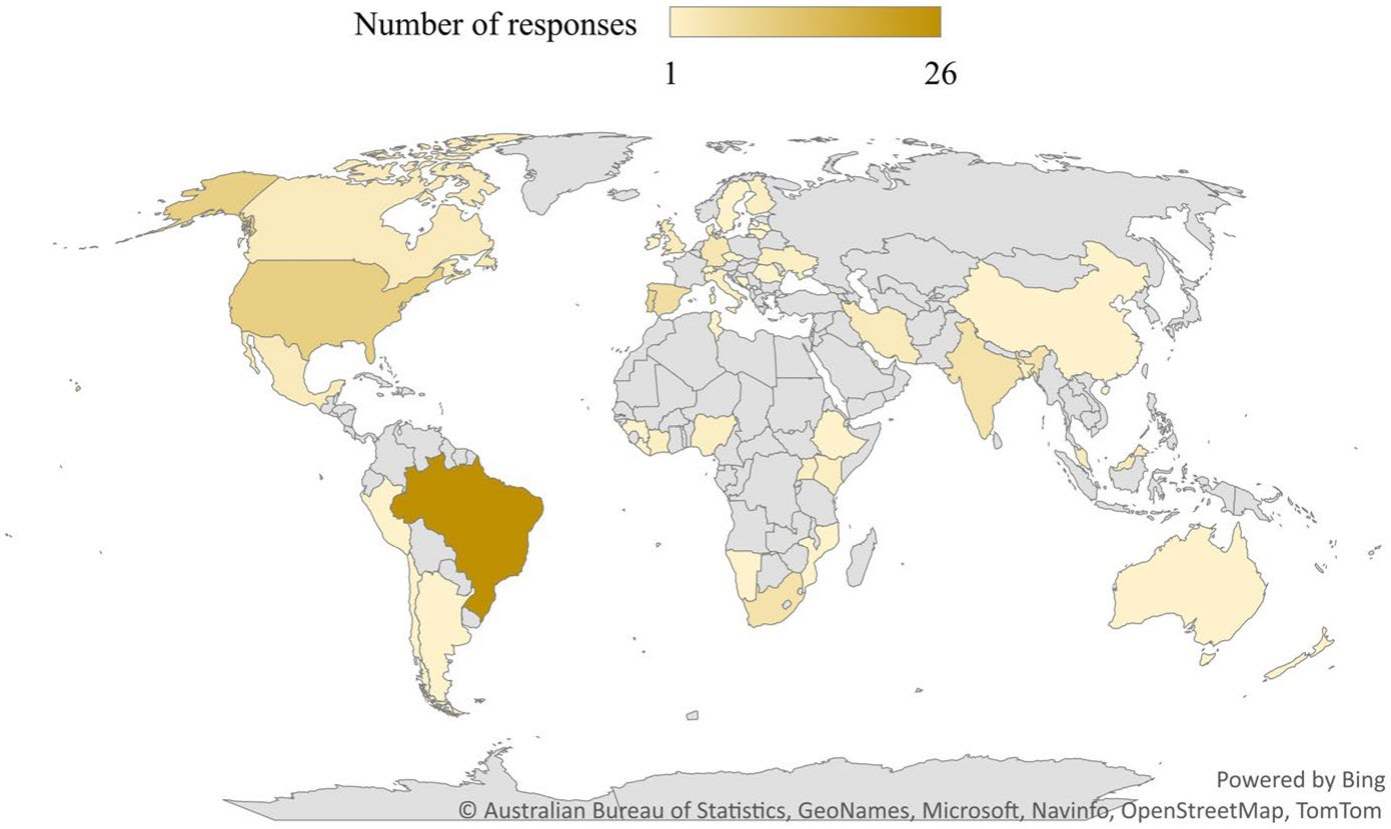
Global distribution of the survey participating countries (*Source* prepared by the authors, Software used: Excel Office 365, v. 2205).

The online survey was applied via Google Forms and disseminated via the international network Inter-University Sustainable Development Research Programme (IUSDRP, https://www.haw-hamburg.de/en/ftz-nk/programmes/iusdrp/)—which gathers over 140 universities worldwide, and the scientific networks to which the co-authors are involved, thus characterizing a convenience sampling strategy. The study collected 120 responses during two weeks, between August–September 2021, and reached global coverage with 45 participating countries on all continents, considered a significant number, as illustrated in Fig. 2.

The internet-based survey ^27–30^, as the method chosen for this study, is a standard procedure, along with qualitative research with interviews and quantitative research with questionnaires and surveys, which requires no specific ethics consent in Germany, as confirmed by the German Association of Medical Ethics Committees. The survey implementation followed the procedures and guidelines used in surveys in Germany and at HAW Hamburg, the lead organisation. Informed consent was sought from all participants, who voluntarily agreed to proceed with the completion of the questionnaire, anonymised so that no personal details were stored. The authors, part of the IUSDRP, regularly publish papers using a similar approach [such as Refs. ^17,31,32^] and the respective journals accept the procedures used. The questionnaire was validated by a group of sustainability experts in the field.

The respondents come from the public (74%) and private institutions (19%), with other options (7%) including non-profit, community, or regional institutions. In terms of enrolment, the institutions represented in the sample cover a wide range of possibilities: 33% from institutions with less than 10,000 students, 27% with enrolment numbers between 10,001 and 20,000, 23% between 20,001 and 40,000, and 18% with over 40,001 students. Almost half of the respondents are educators as their primary role at their institutions (48%), followed by researchers (39%) and colleagues engaged in administrative roles (13%).

The sampling strategy and sample characteristics do not allow for generalizable outcomes, but the different profiles and the international approach make this study unique for filling in the gap in the literature within an exploratory investigation framework on the collective and combined efforts of universities and cities towards sustainable development.

## Results and discussion

According to the perception of the sample on the current level of emphasis given to the relationship between their universities and city organisations on initiatives related to sustainable development, the most common context is cooperation as part of the activities (39%). For 45% of the respondents, the cooperation is a priority or a top priority (23% and 12%, respectively), and for 26% of the respondents, the cooperation is among the most challenging circumstances, from a spectrum of limited cooperation (24%) to no interest at all (2%).

**Figure 3:**
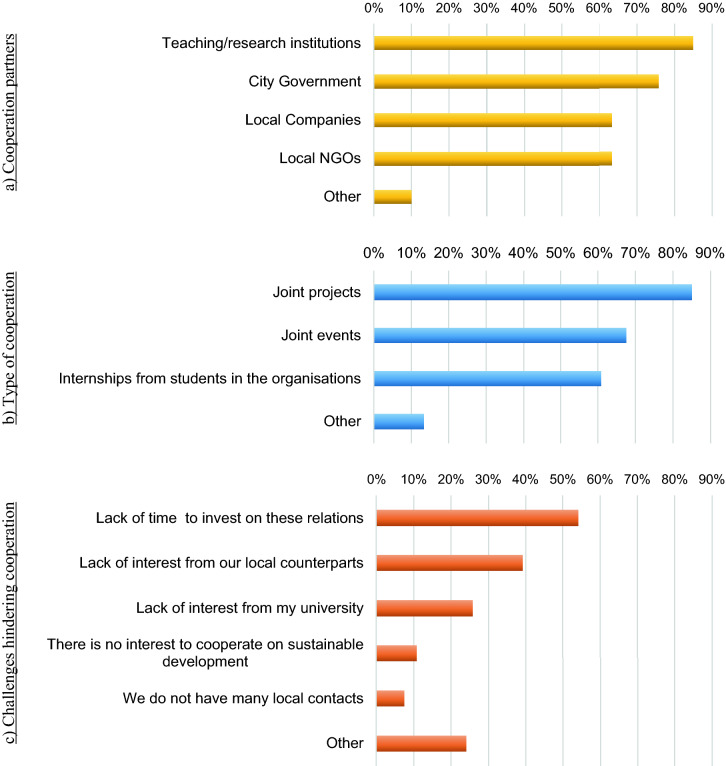
Cooperation partners, type of cooperation, and hallenges hindering cooperation (*Source* prepared by the authors).

Figure 3 summarises the three aspects dedicated to analysing terms of cooperation between the respondents’ universities and city organisations: (a) cooperation partners, (b) type of cooperation, and (c) challenges that hinder cooperation.

Teaching and research institutions seem to be the most common partners addressing sustainable development, selected by 85% of the respondents, followed by the city government, with 76% of the respondents. Local companies and NGOs represented 63% of the sample, while other partners (10% of the respondents) include cooperatives, state and federal government, international organisations (such as the United Nations), local communities, and regional associations.

Collaboration through joint projects is the main strategy reported (85%) followed by joint events (68%) and internships from students in the organisations (61%). Other mentioned means of relationship include community engagement and service-learning activities, agreements for knowledge transfer or sustainable purchasing, memberships, research and teaching activities, guest lectures, technical reports, and participation in municipal councils.

When it comes to challenges that hinder efforts to cooperate on matters related to sustainable development with city organisations, the lack of time to invest in relationships was indicated by 54% of the sample; the lack of interest is also a worrying barrier, both from the local counterparts (39%) and from the universities (26%). Lack of interest and lack of local contacts in cooperation for sustainable development does not seem to represent significant challenges based on the sample perception. On the other hand, lack of funding and resources represented 15% of the total sample; respondents listed several challenges, namely the lack of skills to collaborate properly, lack of political will, the existence of other more urgent matters, risk of corruption on political level impacting the cooperation, greenwashing, complicated governance on local issues, and excessive teaching work and little integration and participation of society.

The examples of synergies presented in Table 1 are illustrative of the in-depth investment that higher education institutions (HEIs) are willing to carry out in order to successfully intervene and inspire the surrounding communities to achieve a higher level of commitment towards sustainable development and the pursuit of the SDGs.

The University of Southern Santa Catarina, Brazil, is invested in acting as a driver of sustainable communities. Accordingly, the study by Amorim et al. ^22^, includes, among other reflections, a project developed in the state of Santa Catarina, the “Water Resources Plan of the Itapocu River Basin”. It is a partnership between the government, the University, and the community, aimed at attaining sustainable collaborative management of the Itapocu basin and its water resources, reducing the water losses and improving the savings, combating the scarcity of water in the region. This example helps to illustrate how important are the actions implemented in the context of the University to promote sustainable development in the region surrounding the campuses, with implications at the local and global level through the investment in specific actions that contribute to saving money and reaching efficiency in public resources, specifically in connection with hydric resources stored in aquifers in the Itapocu river basin to be used to ensure water security in the region, with implications in the environment, e.g., floods, and health, e.g., sewage, of the communities, resulting in a symbiotic prolific effort to advance sustainable development.

More than half of the sample reported a lack of time to invest in relationships. Withycombe Keeler et al. ^23^ underpin that a one-to-one relationship between the city, as well as regions, and the university contributes to a better understanding of how places and contexts shape their sustainability transformation and how to learn from one another. Over the five case studies presented in Table 1, city–university partnerships (CUPs), Withycombe Keeler et al. ^23^ found that the co-creation of a framework to assist in diagnosis—gaps and synergies, strategy development, continuous learning, and research, strategy transfer, and scaling, promoted a common language in terms of goals, approaches, and solutions in a systematic way for sustainability. Moreover, the framework represents a useful tool for continuous learning and transfer, supporting the capacity building in the city, for example, joint research, joint projects, and student experience. For strengthening the effectiveness of the capacity building of CUPs, Withycombe Keeler et al. ^23^ propose to invest in bridge-building with students as interns in the city; a networking platform to elevate evidence and provide legitimacy; educational and research activities to develop engaged teams; resilience building in expertise and relationships; and a transparent flow of information.

As stated by Iwaniec et al. ^24^, urban planners and decision-makers tend to focus on more easily applicable solutions for sustainability challenges, even though these require further thinking and planning. This represents an opportunity for universities to collaborate, e.g., by means of joint events or projects, the most common types of cooperation. Additionally, the approach of establishing a governance committee with members from HEIs, industry, and the community in the context of university towns ^25^ could also be useful in broader contexts, as a strategy to foster symbiotic collaborations between universities and cities to promote sustainable development. This strategy could be valuable to overcome the most common challenges of lack of time and interest in these relations, as responsibilities and resources could be shared, assuring that managing sustainable development solutions to be implemented in practice, besides being both pro-environmental and pro-social, are profitable for involved stakeholders ^33^.

## Conclusions: towards more symbiotic relations

Over the past years, cities have been seeking partnerships with universities due to their ability to accelerate complex problem-solving, thus enhancing the transformative capacity needed to achieve sustainable development. CUPs that focus on capacity-building for sustainability transformations, for instance, may help staff and students to develop the skills and test innovative interventions for sustainability solutions, bring passion and funding to governmental institutions, and provide space for creativity and adaptability ^19^. As a result, through cooperation, empowerment is achieved in both universities and society.

The relevance of this study does reside in the fact that it points out to a demanding need for greater integration of universities and cities in undertaking symbiotic joint efforts in the context of advancing sustainable development, as demonstrated by the results obtained.

Despite some limitations, such as non-identification of the cities, due to anonymity, the implications of this paper are twofold. Firstly, since cities across the globe have been partnering with universities, there is a significant potential for successful cooperation on sustainable development. Secondly, it shows the potential of the co-creation of new knowledge and co-management of projects, which has proven successful in many cases.

In order to be able to take full advantage of the many opportunities that cooperation in sustainable development may offer, some policy recommendations must be considered:

- Formal cooperation agreements between cities and local universities to work together in the field of sustainable development.
- Funding provisions in cities’ budgets to support cooperation.
- Definition of key sustainability topics to which a focus may be given.
- Provisions for mutual staff exchanges within both types of organisations.

Additionally, it is important that universities should work closely with organisations in their regions, making them relevant to their communities. In other words, more symbiotic relations are needed across the globe. These include joint projects and events involving academics on the one hand, and local decision-makers, industry, and local stakeholders on the other. Also, and bearing in mind the potential contribution of students, they should be encouraged to work more closely with actors at the local and regional levels on matters related to sustainability, which may vary from sustainable production to transport and consumption. By doing so, they can learn about how to handle social challenges, a valuable complement to their university education. Finally, to yield the expected benefits, the right framework conditions must be created so that academic staff is encouraged to take better account of local and regional issues, as part of their teaching and research programmes, a major sustainable development worldwide challenge.

## Data Availability

The datasets generated during the current study are available from the corresponding author on reasonable request.
