# Author Correction: Towards symbiotic approaches between universities, sustainable development, and cities

**DOI:** 10.1038/s41598-023-27833-8

**Published:** 2023-01-11

**Authors:** Walter Leal Filho, Liliana Caughman, Maria Alzira Pimenta Dinis, Fernanda Frankenberger, Anabela Marisa Azul, Amanda Lange Salvia

**Affiliations:** 1 Department of Natural Sciences, Manchester Metropolitan University, Chester Street, Manchester, M115GD UK; 2 Research and Transfer Centre “Sustainable Development and Climate Change Management”, Hamburg University of Applied Sciences, Ulmenliet 20, 21033 Hamburg, Germany; 3 Postdoctoral Research Scholar, Earth Systems Science for the Anthropocene, Arizona State University, Tempe, USA; 4 UFP Energy, Environment and Health Research Unit (FP-ENAS), University Fernando Pessoa (UFP), Praça 9 de Abril 349, 4249-004 Porto, Portugal; 5 Business School PUCPR, Pontifical Catholic University of Paraná, Rua Imaculada Conceição, Curitiba, 1155 Brazil; 6 Brazil Business School, Universidade Positivo, Rua Pedro Viriato Parigot de Souza, Curitiba, 5300 Brazil; 7 CNC-Center for Neuroscience and Cell Biology, University of Coimbra, 3004-504 Coimbra, Portugal; 8 CIBB-Center for Innovative Biomedicine and Biotechnology, University of Coimbra, Coimbra, Portugal; 9 IIIUC-Institute for Interdisciplinary Research, University of Coimbra, 3030-789 Coimbra, Portugal; 10 Graduate Program in Civil and Environmental Engineering, University of Passo Fundo, Campus I - BR285, São José, Passo Fundo, RS 99052-900 Brazil; 11 European School of Sustainability Science and Research, Hamburg University of Applied Sciences, Ulmenliet 20, 21033 Hamburg, Germany

Correction to: *Scientific Reports* https://doi.org/10.1038/s41598-022-15717-2, published online 06 July 2022

The original version of this Article omitted an affiliation for the corresponding author Amanda Lange Salvia.

The correct affiliations are listed below:

Graduate Program in Civil and Environmental Engineering, University of Passo Fundo, Campus I - BR 285, São José, Passo Fundo, RS, 99052-900, Brazil

European School of Sustainability Science and Research, Hamburg University of Applied Sciences, Ulmenliet 20, D-21033, Hamburg, Germany

The original Article has been corrected.

